# Supplementary material for: The clinical association of programmed death-1/PD-L1 axis, myeloid derived suppressor cells subsets and regulatory T cells in peripheral blood of stable COPD patients
Source: PeerJ. 2024 Mar 27;12:e16988. doi: 10.7717/peerj.16988 (PMC10981408; doi:10.7717/peerj.16988)
Supplement: Supplemental Information 2 [file peerj-12-16988-s002.docx]

**Supplement Table1. Antibody for FACS analysis**

| Antibody | Company | Catalog number | Dosage |
| --- | --- | --- | --- |
| anti Human-CD3 | eBioscience | MHCD0327 | 10μL/100μL test sample |
| anti Human-CD4 | eBioscience | 25-0049-42 | 10μL/100μL test sample |
| anti Human-CD25 | eBioscience | 12-0257-42 | 10μL/100μL test sample |
| anti Human-CD127 | eBioscience | 17-1278-42 | 10μL/100μL test sample |
| anti Human-CTLA-4 | eBioscience | 85-46-1529-42 | 10μL/100μL test sample |
| anti Human-PD-1 | eBioscience | 61-2799-42 | 10μL/100μL test sample |
| anti Human-CD11B | eBioscience | 46-0118-42 | 10μL/100μL test sample |
| anti Human-CD14 | eBioscience | 61-0149-42 | 10μL/100μL test sample |
| anti Human-CD33 | eBioscience | 56-0338-42 | 10μL/100μL test sample |
| anti Human-CD15 | eBioscience | 11-0159-42 | 10μL/100μL test sample |
| anti Human-CD45 | eBioscience | 47-0459-42 | 10μL/100μL test sample |
| anti Human-HLA-DR | eBioscience | 25-9952-42 | 10μL/100μL test sample |
| anti Human-PD-L1 | eBioscience | 85-12-5888-42 | 10μL/100μL test sample |
| anti Human-PD-L2 | eBioscience | 25-9952-42 | 10μL/100μL test sample |
